# Annotations

**Published:** 1897-07-17

**Authors:** 


### ANNOTATIONS.

### Midwifery Nurses.

Many medical men appear to be in favour of giving to midwives the title of midwifery nurses, while others look upon the name as a mere trivial detail. It may be worth while, however, to bear in mind that it is a much more serious matter to the medical profession to allow midwifery nurses to work independently, only sending for help when in difficulty, than to allow midwives to do so. The following paragraph from *Nursing Notes* is worthy of careful consideration by those medical men who are advocating the use of the hybrid title: "We much wonder what the inauguration of this new class of nurse will lead to. The partial independence of the midwife is well understood, but when the public grasps the fact that it is permitted for obstetric nurses to attend confinements without a doctor they will see no reason why surgical, medical, mental, and gynæcological nurses should not also dispense with the doctor's supervision, and only send for him when they get a little fogged, and we fear there will be nurses to meet this demand. It has always been against the professional ethics for *nurses* not to be directly under medical direction, but if the obstetric nurse is free, why not her medical and surgical sister? We offer this for the consideration of the medical profession."

### The Ship Surgeon.

To be a ship surgeon is the ambition of many a good man, who yet shows after a voyage or two how uncongenial to his temperament have been the petty details of the life. To those, however, who are adaptable, and can derive pleasure even from queer surroundings, a few sea voyages give an admirable finish to a medical education, for it cannot be denied that the constant grind for examinations and the careful following of a rigid curriculum tend to make a man narrow, however skilful he may by such means become. Dr. Maidlow wrote an admirable article in the *St. Bartholomew's Hospital Gazette* some time ago which well illustrated the sort of temper in which one should meet the difficulties which must arise in that as in every sort of work. It has to be recognised that the life offers something better than a struggle for the fleshpots and a comfortable berth. If discomforts are met with, "they are counteracted by the sight of Gibraltar and all the interest of the East, and the joy of seeing plague and beri-beri, ague, &c.," and if a man does not feel that these joys will counteract discomforts — well, he had better not go to sea. Contrary to what many people think, the life of a ship surgeon on board one of the great steamers is far from being an idle one. With 400 passengers and a crew of about 300 there is always more or less illness, besides the work which arises from sea sickness in the early days of every voyage. Then, even if the work should not be heavy, an accident is always on the cards; a fireman may get his fingers crushed, or there may be a sunstroke, or a fight, or some doubtful rash may occur which may require measures for isolation, or, as happens sometimes from violent sea sickness, a hernia may descend and may require assistance. Then there are the children, and travelled children are very precocious; and the mothers, who, of course, know everything! Then again, there are the knowing ones, who prescribe for themselves and send round for chlorodyne or for some huge dose of quinine, as if one kept a chemist's shop. But these people have often been through much, and have had experience of many climes, of many diseases, and also of many remedies; and, if the young doctor is receptive, even from these people, exacting as they may seem, he may learn much. The responsibility thrown on the ship surgeon is often very considerable, especially when on arriving at a port he has to meet the health officer at the gangway and declare on oath the sanitary condition of the ship, when perchance there may be a doubtful case on board. "It is all very well to talk of the happiness of doing one's duty; sometimes it causes much unhappiness." The life, however, is very interesting while the interest lasts; and when it lasts no longer then the ship surgeon had better look out for a berth ashore. But all his life he will be a better fellow for his time at sea, and all his life, even to the last, he will have his memories to fill many a dull hour.

### American Health Resorts.

It is to be feared that medical men practising at this side of the Atlantic have but a very imperfect acquaintance with even the names of the very numerous health resorts which are to be found in North America. The *Medical Record* of New York has published a special number containing short descriptions of upwards of a hundred of these places, from which we can judge of the very large variety of climatic resorts our American cousins have at their disposal. It is very apparent from the stress laid upon coolness as a dominant attraction how great is the heat of the summer in the States. Watering-places seem to dot the northern shores both on the Atlantic and Pacific coasts, while both in New England and New York there are mountains, lakes, and rivers which are unsurpassed in the variety of their attractions. For those also who wish for the bracing effects of high altitudes plenty of places are available in the region of the Rocky Mountains.

### Medical Certificates and Workmen's Compensation.

The discussion on the Workmen's Compensation Bill which took place in the House of Commons on the third reading shows again what an important part medical men will be asked to take in its administration. The decision of all questions as to the amount of compensation will lie with the arbitrator, who will have the services of an officially-appointed medical assessor. But in regard to the weekly payments it has been arranged that "any workman claiming compensation under this Act shall, if so required by the employer, from time to time submit himself for examination by a duly qualified medical practitioner provided and paid for by the employer. If the workman refuses to submit himself to such examination, or otherwise obstructs the same, his right to such weekly payments shall be suspended until such examination has taken place." We hope it will be recognised both by the workmen and by the masters how peculiarly responsible and anxious a matter this sort of examination will be in many cases, and that it will not be allowed in deference to any penny-wise ideas of economy, to drift into a matter of form.

